# SCFAs Ameliorate Chronic Postsurgical Pain–Related Cognition Dysfunction via the ACSS2-HDAC2 Axis in Rats

**DOI:** 10.1007/s12035-022-02971-8

**Published:** 2022-07-28

**Authors:** Zhen Li, Tianning Sun, Zhigang He, Zhixiao Li, Wencui Zhang, Jie Wang, Hongbing Xiang

**Affiliations:** 1 Department of Anesthesiology and Pain Medicine, Tongji Hospital of Tongji Medical College, Huazhong University of Science and Technology, Wuhan 430030, Hubei, China; 2 Key Laboratory of Magnetic Resonance in Biological Systems, State Key Laboratory of Magnetic Resonance and Atomic and Molecular Physics, Innovation Academy for Precision Measurement Science and Technology, National Center for Magnetic Resonance in Wuhan, Wuhan Institute of Physics and Mathematics, Chinese Academy of Sciences-Wuhan National Laboratory for Optoelectronics, Wuhan, 430071 Hubei China; 3 University of Chinese Academy of Sciences, Beijing, 100049 China

**Keywords:** Chronic postsurgical pain, Cognitive function, Short-chain fatty acids, Gut microbiota, Histone acetylation, Synaptic transmission

## Abstract

Patients with chronic postsurgical pain (CPSP) frequently exhibit comorbid cognitive deficits. Recent observations have emphasized the critical effects of gut microbial metabolites, like short-chain fatty acids (SCFAs), in regulating cognitive function. However, the underlying mechanisms and effective interventions remain unclear. According to hierarchical clustering and 16S rRNA analysis, over two-thirds of the CPSP rats had cognitive impairment, and the CPSP rats with cognitive impairment had an aberrant composition of gut SCFA-producing bacteria. Then, using feces microbiota transplantation, researchers identified a causal relationship between cognitive-behavioral and microbic changes. Similarly, the number of genera that generated SCFAs was decreased in the feces from recipients of cognitive impairment microbiota. Moreover, treatment with the SCFAs alleviated the cognitive-behavioral deficits in the cognitively compromised pain rats. Finally, we observed that SCFA supplementation improved histone acetylation and abnormal synaptic transmission in the medial prefrontal cortex (mPFC), hippocampal CA1, and central amygdala (CeA) area via the ACSS2 (acetyl-CoA synthetase2)-HDAC2 (histone deacetylase 2) axis. These findings link pain-related cognition dysfunction, gut microbiota, and short-chain fatty acids, shedding fresh insight into the pathogenesis and therapy of pain-associated cognition dysfunction.

## Introduction

Chronic postsurgical pain (CPSP), frequently comorbid with cognition impairments such as learning and memory [1–3], has long piqued the interest of patients and medics due to its high incidence (approximately 10–50%) and deleterious effect on patients [4]. Conversely, cognitive functions can influence pain [5–7]. The mechanisms of chronic postsurgical pain-induced cognitive dysfunction are extremely complicated, and effective interventions remain unclear. The attentional cost hypothesis, brain plasticity, and structural changes have been proposed thus far [8].

Evidence [9, 10] has highlighted the importance of gut microbial metabolites in regulating cognitive function via regulation of the blood–brain barrier permeability [11], brain energy homeostasis [12], and synaptic transmission [13]. During anaerobic fermentation, the microbiota’s major products are host-absorbable short-chain fatty acids (SCFAs), including acetate and butyrate, which are the most vital and pleiotropic functional components of host-microbe crosstalk [14]. SCFAs have been shown to have promising therapeutic benefits in various neurological disorders, including autism [15], diabetes-induced cognitive impairment [10], and maternal obesity–induced cognitive dysfunctions [9]. However, whether SCFAs restore cognitive behavioral deficits in chronic postsurgical pain rats is uncertain.

Histone acetylation epigenetic modifications have been implicated in synaptic plasticity, learning, and memory. Specifically, histone deacetylase 2 (HDAC2) has been shown to regulate cognition formation negatively and is likely to be the most relevant isoform responsible for HDAC inhibitor–induced memory enhancement [16]. Upregulation of HDAC2 activity has been associated with reduced dendritic spine density, synapse number, and expression of several genes involved in learning and memory [17, 18]. Notably, sodium butyrate, an HDAC inhibitor, improved learning capacity even after massive neuronal loss [18] and recovered memory deficits by enhancing histone acetylation [19]. Using ^13^C-carbon tracing in conjunction with acetyl-proteomics, researchers discovered that even in the presence of excess glucose, lipid-derived acetyl-CoA was a major (up to 90%) source of carbon for histone acetylation and may affect the epigenome [20]. Acetyl-CoA synthetase2 (ACSS2), which employs acetate as a source of acetyl-CoA for nuclear synthesis [21], is a direct controller of histone acetylation and spatial memory in mammals. Attenuation of ACSS2 expression in adult mice affects spatial memory while simultaneously increasing the expression of memory-related neuronal faulty genes pre-bound by ACSS2 [22].

Given these findings linking gut microbiota and metabolism to histone acetylation and cognitive function, we conducted a comprehensive study to investigate the impact of SCFAs (acetate and butyrate) on chronic pain-related cognitive dysfunction and underlying mechanisms in skin/muscle incision and retraction (SMIR) rats. Here, we report that (1) chronic postsurgical pain causes cognitive behavioral deficits in rats, (2) the gut SCFA-producing microbiome mediates pain-related cognitive impairments, and (3) SCFA consumption alleviates chronic postsurgical pain–induced cognitive deficits by improving histone acetylation and synaptic transmission via the ACSS2-HDAC2 axis. Our results may offer a more detailed understanding of how SCFAs alleviate pain-related cognitive deficits, providing more therapeutic strategies for pain-related cognitive dysfunction.

## Materials and Methods

### Experiment Animals

Male Sprague–Dawley (SD) rats (age: 6 weeks) were obtained from the Animal Centre of Tongji Hospital and housed under standard conditions (22 ± 2 °C, 12-h light/dark cycle), with free access to food and water ad libitum. The rats were randomly assigned to different groups. The protocol of all experiments was followed as per the Guide for the Animal Care and Use Committee, Tongji Hospital, and complied with the Guide for the Care and Use of Laboratory Animals (the National Institutes of Health guidelines).

### Behavioral Test

All behavioral tests were completed in awake, unrestrained, and habituated rats. The extent of mechanical sensitivity was determined to evaluate whether SMIR surgery evoked mechanical hypersensitivity. The open-field test was applied to assess locomotor activity. The Y-maze test was used to determine the spatial orientation learning memory ability, and the novel object preference test was performed to evaluate the non-spatial visual learning memory [23–25]. The test devices were cleaned with 75% ethanol after every trial to avoid the interference of any odor.

### Assessment of Mechanical Sensitivity

As described previously, mechanical sensitivity was measured based on the paw withdrawal threshold (PWT) [26, 27]. Before conducting the test, the rats were allowed to acclimate for > 30 min in separate plastic chambers over a mesh platform. Then, according to the up-down paradigm, the filaments of sequentially increasing stiffness (1.0–15 g) were vertically applied to the suitable plantar surface until the filament bent. Rapid withdrawal or licking of the paw was defined as a positive response.

### Open Field Test

After habituation, each rat was placed into a black open field chamber (100 × 100 × 40 cm^3^) to move spontaneously for 5 min. The locomotor activity was measured by the total distance travelled [23].

### NOP

The test was completed in a black open field in two steps. After accommodation without objects, the rat had free access to the two identical objects pasted on the symmetrical positions of the open field for 5 min at the training stage. After 2 h, the rats were placed again in the same apparatus and allowed to freely explore the same for 5 min with one of the identical objects being replaced with a novel object (test session). The time spent exploring the familiar object (F), and the novel object (N), was recorded. The recognition index was applied to assess the memory function [24, 28].

### Y-Maze Test

The Y-maze was performed with two stages in a Y-shaped maze with three identical arms, angled at 120°. The randomly named start arm (animal entry) and the common arm were always kept open. The new arm was blocked on the first trial, on which the rat explored the two arms for 5 min. After 2 h, all the arms were opened, and the rat had free access to the three arms for 5 min (test trial). Preference for a new arm revealed the short-term spatial reference memory [23, 29].

### SMIR Model

After anesthesia with pentobarbital sodium (50 mg/kg, intraperitoneal), the rats were laid on the back, and their right medial thigh was shaved [30]. After sterilizing the shaved skin, a 1.5–2.0-cm incision, approximately 4–5 mm medial to the saphenous vein, was made in the medial thigh to reveal the muscle and vessels of the thigh. Then, a 1.0-cm incision, approximately 3.0 mm proximal to the saphenous nerve, was made in the superficial muscle layer. Finally, the superficial skin and the muscle were parted to mimic the clinical scenario to allow a retractor to insert into the incision site for retracting (2.0 cm) for 1 h.

### 16S rRNA Microbiome Sequencing

After the behavioral tests, the fecal samples were collected and stored at − 80 °C until DNA extraction [9]. The 16 S rRNA sequencing of the microbiota was performed at the Majorbio Bio-pharm Technology Co., Ltd. (Shanghai, China). After extraction, the total genomic DNA was quantified on 1% agarose gel. The V3–V4 region of the bacterial 16S rRNA was amplified by the universal primers: 338 F and 806R with the GeneAmp® 9700 PCR thermocycler (ABI, USA). After 2% agarose gel extraction, the PCR product was purified and quantified by the AxyPrep DNA Gel Extraction Kit and the Quantus™ Fluorometer, respectively. The adapter sequences were added to the purified amplicons, and paired-end sequencing was performed on the Illumina MiSeq instrument (Illumina, USA). The free Majorbio Cloud Platform (http://www.isanger.com/) was used to perform statistical analyses. Sequences with a similarity cut-off of 97% were grouped into operational taxonomic units (OTUs) (Uparse v7.0.1090).

### Serum SCFA Assay

The concentrations of SCFAs were detected by gas chromatography [9]. For this, 25% metaphosphoric acid (100 mL) was mixed with a serum sample (200 mL) to remove the solid particles. After centrifugation, 4 mL of crotonic acid was added to the supernatants (200 mL), and the mixture was vortexed and then filtered. The SCFAs was separated and detected by gas chromatography spectrometry (Agilent Technologies 7820 GC system).

### Microbiota Depletion and FMT

To generate the pseudo-germ-free animal model, we provided the experimental rats with vancomycin hydrochloride (500 mg/L), ampicillin sulfate (1 g/L), neomycin sulfate (1 g/L), and metronidazole (1 g/L) in their sterile drinking water for 2 weeks [9, 31]. Each mixed fecal sample (1 g) from different unsusceptible or susceptible rats was suspended in 10 mL of sterile phosphate-buffered saline and vigorously vortexed for 5 min. The suspensions were obtained after homogenization and centrifugation to colonize pseudo-germ-free rats [9, 31]. Regarding the European Consensus Conference for fecal microbiota transplantation in clinical practice [32, 33], 0.43 g of feces/kg body weight is recommended, and, accordingly, a 200 g rat should receive 0.86 g feces diluted in 0.86 mL PBS via gavage once a day for 2 consecutive weeks.

### SCFA Supplementation

For the S-AA group, acetate (135 mM) [9] was dissolved in the drinking water supplied to the rats soon after the SMIR surgery until the last day for behavioral tests. For the S-BTA group, butyrate was dissolved in saline and intraperitoneally injected at a dose of 1.2 g/kg 24 h before behavioral tests [19]. The acetate and the butyrate were supplemented in sequence for the S-AA-BTA group.

### Electrophysiology Recording in Brain Slices

As previously described [26, 27, 34], the whole-cell recordings in vitro from the medial prefrontal cortex (mPFC), hippocampal CA1, and central amygdala (CeA) neurons were used to examine the alterations of the synaptic transmission after SCFA treatment. To prepare the slices, we immediately immersed the brain into the cold oxygenated cutting solution after anesthesia and sliced the brain into a thin Sect. (300 μm thick) with a vibratome (VT1200S; Leica, Germany). After incubating for at least 1 h at 30 °C in oxygenated artificial cerebrospinal fluid (ACSF), an individual slice was placed in the submersion-recording chamber and continuously perfused with ACSF (2 mL/min). The ACSF consisted of (in mM): 125.0 NaCl, 1.2 NaH_2_PO_4_, 26.0 NaHCO_3_, 5.0 KCl, 2.6 CaCl_2_, 1.3 MgCl_2_, and 10.0 glucose. Only one neuron was recorded in each slice.

Data acquisition and analysis of the electrical signals were performed with the Axonpatch 700B Amplifier (Molecular Devices, USA), the Digidata 1500B Digital Converter (Molecular Devices, USA), and the pClamp 10 software (Molecular Devices, USA). Excitatory pyramidal neurons and inhibitory interneurons in different regions were identified by accommodating responses to a sustained depolarizing intracellular current stimulation. Regular spiking neurons with a firing rate < 5 Hz were believed to correspond to the excitatory pyramidal neurons versus fast-spiking neurons (firing rate > 10 Hz) with shorter duration waveforms believed to correspond to the inhibitory interneurons [35, 36]. The WPI recording pipettes (4–7 MΩ, USA) were filled with the pipette solution containing (in mM) 122.0 potassium-gluconate, 2.0 MgCl_2_, 0.3 CaCl_2_, 5.0 NaCl, 5.0 EGTA, 10.0 HEPES, 4.0 Mg-ATP, and 0.3 Na_3_-GTP (pH 7.30 and 300 mOsm/kg). The miniature excitatory postsynaptic currents (mEPSCs) were recorded at − 70 mV with picrotoxin (100 μM) and tetrodotoxin (TTX) (1 μM). A fixed 5-min length of traces was then analyzed.

### Immunostaining

The experimental animals were transcardially perfused with PBS and paraformaldehyde, and their brain tissues were post-fixed in 4% paraformaldehyde/phosphate-buffered saline at 4 °C. Then, the tissues were cut into Sects. (20 mm) and stored at − 20 °C until immunostaining. After washing with PBS, the sections were fixed and permeabilized with 0.3% Triton and blocked with 5% goat serum in 1 × PBS buffer. These sections were then incubated with the rabbit anti-ACSS2 (1:100) or the rabbit anti-HDAC2 (1:100) at 4 °C overnight. After washing with the PBS buffer, the sections were treated with CY3 goat anti-rabbit (1:200; Jackson) secondary antibodies at room temperature for 2 h. Subsequently, the sections were treated with 4′,6-diamidino-2-phenylindole (DAPI) (nuclear staining, 1:8000) for 8 min. We then imaged the tissue sections with the OLYMPUS Slice Scanning System (6C08534, TOKYO, Japan).

### Western Blotting

Following brain removal after deep anesthetization with sodium pentobarbital, the mPFC, hippocampus, and CeA were homogenized in lysis buffer. After centrifugation, the protein concentration of the supernatant was detected by using the bicinchoninic acid kit (Boster). Then, 20 μg of each nuclear protein sample was separated on 10% SDS-PAGE gels and transferred onto a polyvinylidene fluoride membrane, which was then blocked in 5% skim milk for 2 h at room temperature, and the membrane was incubated overnight at 4 °C with different primary antibodies. Then, the blots were washed thrice and incubated with the secondary antibody for 1 h at room temperature. Immunoreactivity was quantified by densitometry with enhanced chemiluminescence.

The following primary antibodies were used: anti-GAPDH (1:1000, A19056, ABclonal); anti-ACSS2 (1:1000, T55995, Abmart); anti-HDAC2 (1:1000, 57156S, CST); anti-Ace-H4K12 (1:1000, 13,944, CST); anti-Ace-Lysine (1:1000, 9441, CST).

### Statistical Analysis

All data were reported as the mean ± SEM. The normality distribution assessment was evaluated by the Kolmogorov–Smirnov test, and differences among the groups were assessed by one-way analysis of variance (ANOVA), followed by Tukey’s test. The significance testing was two-tailed, and *p* < 0.05 was statistically significant. The rats that underwent the SMIR surgery were divided into the susceptible group (cognitively compromised pain rats) and the unsusceptible group (cognitively normal pain rats) based on the Ward method and Euclidean distance square as distance measurement using the hierarchical cluster analysis of the data from the novel object recognition test and the Y-maze test [31]. Statistical analyses and graphs (except the 16S rRNA microbiome sequencing analyses) were performed with the Adobe Illustrator Artwork 14.0 and GraphPad Prism 8.0.

## Results

### Chronic Postsurgical Pain Rats Displayed Cognitive Behavioral Deficits

**Fig. 1:**
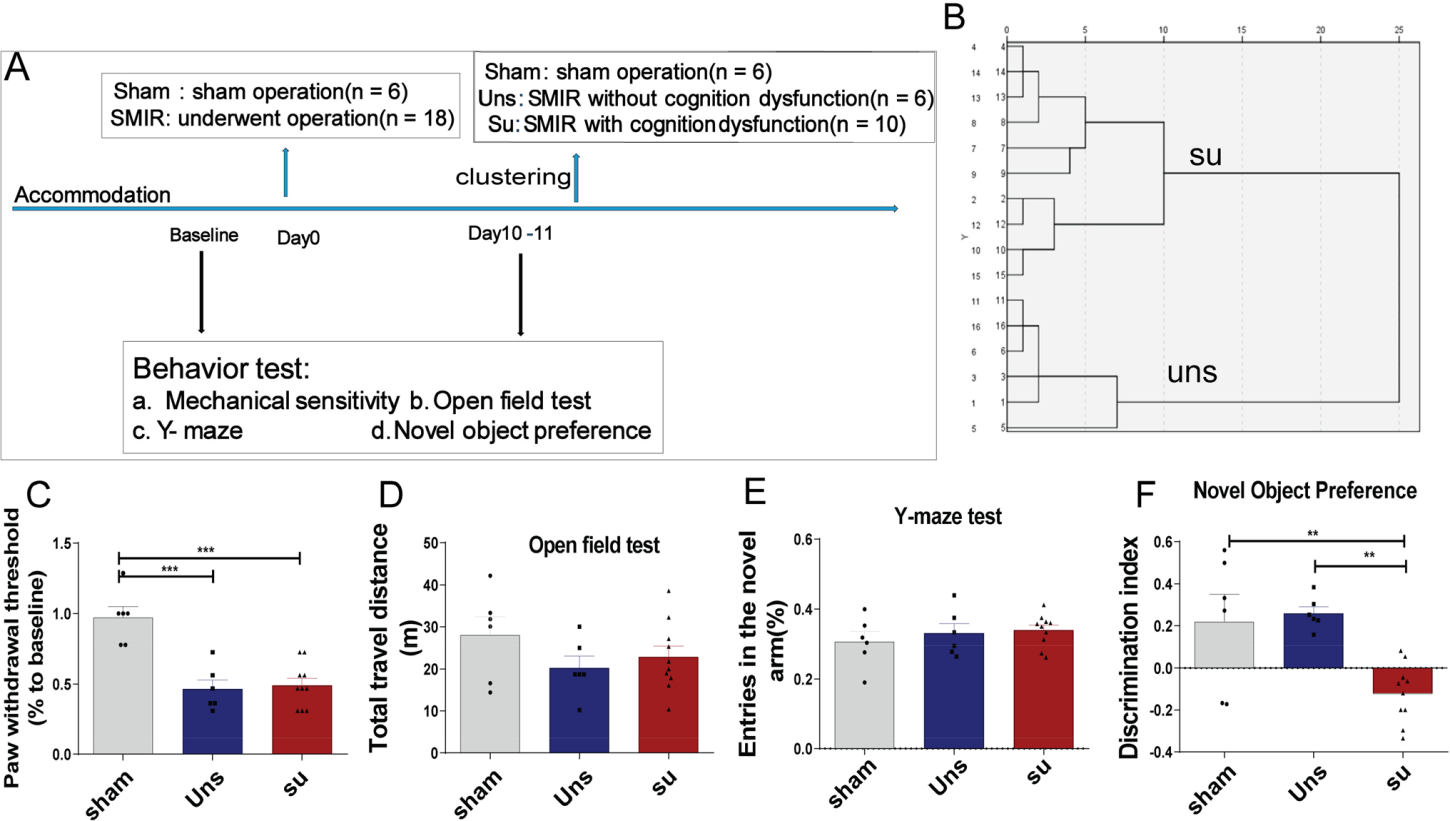
Chronic postsurgical pain–induced significant mechanical hypersensitivity and cognitive impairment. **A** Schematic illustration of the experimental design. **B** Dendrogram of hierarchical clustering analysis after SMIR surgery. Rats who underwent SMIR surgery were divided into su group (*n* = 10, cognitively compromised pain rats) and uns group (*n* = 6, cognitively normal pain rats) by hierarchical clustering analysis on the Y-maze test and the novel object preference test. Behavioral tests after SMIR surgery, including **C** mechanical sensitivity, **D** the open field test, **E** the Y-maze test, and **F** the novel object preference test. Tukey’s post hoc tests; **p < 0.01, ***p < 0.001, ****p < 0.0001

Using a hierarchical clustering analysis on cognitive function, the CPSP rats were divided into two groups: unsusceptible (no cognitive damage) (*n* = 10) and susceptible (cognitive impairment) (*n* = 6) 10 days after SMIR surgery (Fig. 1A and B). PWL (paw withdrawal threshold) in rats was dramatically reduced by SMIR surgery (Fig. 1C), although no motor impairment was seen (Fig. 1D). There was no significant difference in the Y-maze test (Fig. 1E). The susceptible rats performed poorly on the cognitive behavior test due to impaired non-spatial visual learning memory (Fig. 1F). According to these findings, SMIR surgery caused severe mechanical hypersensitivity and non-spatial memory problems in rats.

### CPSP Rats with Cognitive Impairments Showed Abnormal Gut Microbiota Composition

**Fig. 2:**
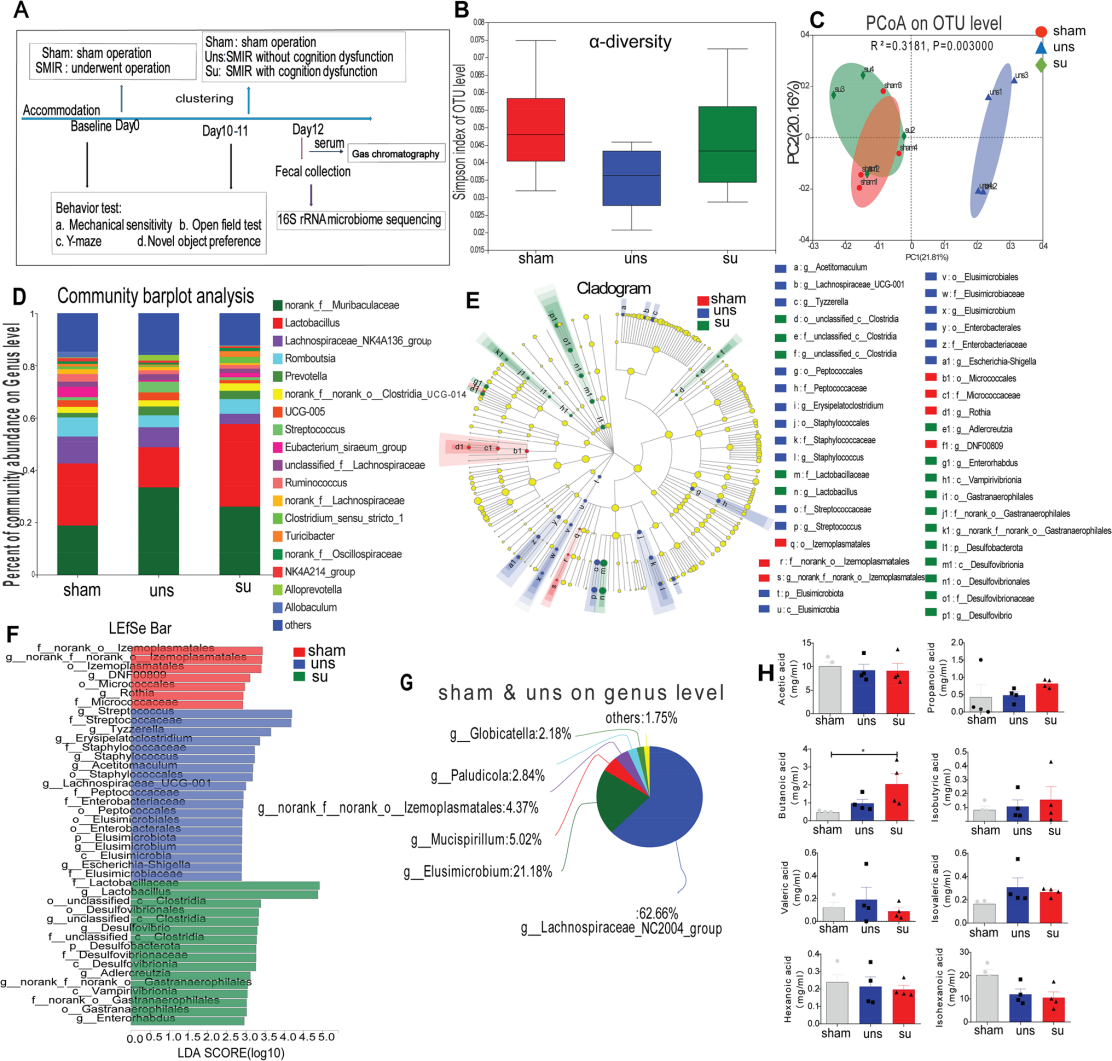
The CPSP rats with cognitive impairment showed abnormal gut microbiota composition. **A** Schematic illustration of the experimental design. **B** Simpson index. **C** Principal coordinate analysis (PCoA). **D** The relative abundance of bacteria at the genus level. All genera with an average relative abundance below 1% were grouped to “others.” **E**–**F** Linear discriminant analysis effect size (LefSe) analysis: **E** cladogram and **F** linear discriminant analysis (LDA); red indicates clades enriched in the sham group, blue indicates clades enriched in the uns group (cognitively normal pain rats), whereas green indicates clades enriched in the su group (cognitively compromised pain rats). **G** Common species in the sham and uns group at the genus level. **H** SCFA concentrations in serum

Metabolites produced by gut bacteria have influenced cognitive function [14]. As a result, 16S rRNA gene sequencing was used to analyze the changes in gut microbiota composition following SMIR (Fig. 2A). While bacterial α-diversity did not differ between the three groups (Fig. 2B), the β-diversity (PCoA, *R*^2^ = 0.3181, *p* = 0.003; Fig. 2C) showed notable differences between the three groups, which revealed that pain induced by SMIR or lower cognition induced by pain altered the microbiota composition. In addition, the microbiome structure was considerably altered after SMIR in rats (Fig. 2D and G), and the quantity of SCFAs-producing bacteria [37, 38], particularly *Lachnospiraceae* and *Elusimicrobium*, was decreased in the susceptible group (Fig. 2E−G).

Given the scarcity of SCFA-producing genera in the susceptible group, we looked at SCFAs in the serum of the three groups. Surprisingly, the sensitive rats had greater butyrate concentrations than the sham rats, despite no significant change in other SCFAs across groups (Fig. 2H). These findings suggested that the number of gut bacteria was strongly connected to cognitive impairments.

### Microbiota Transplantation Induced Cognitive Impairment Behaviors and Reshaped the Gut Microbiome

**Fig. 3:**
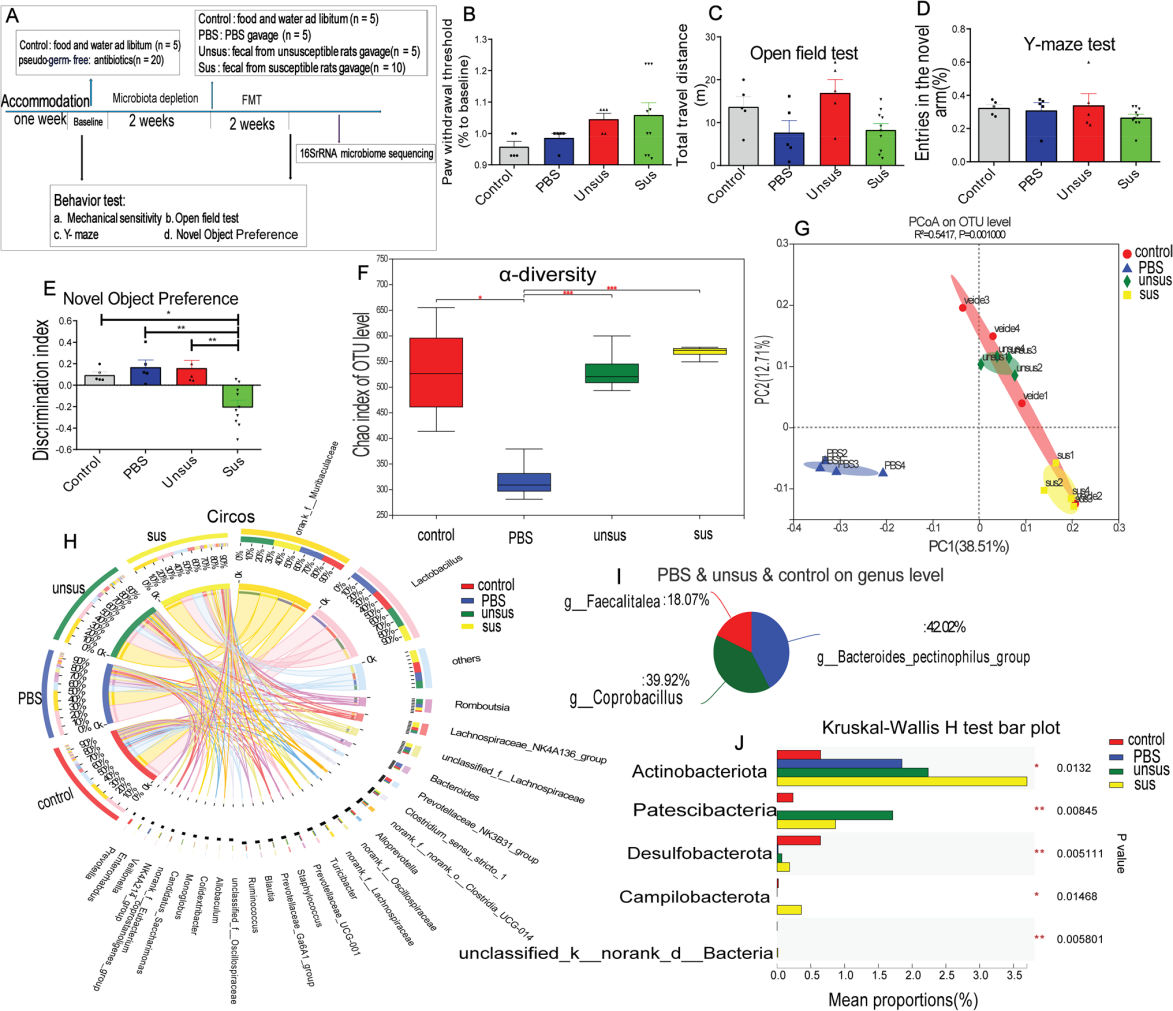
Microbiota from CPSP rats with poor cognition induced cognitive impairment behavior and induced abnormal gut microbiota composition. **A** Schematic illustration of the experimental design. Effect of microbiota transplantation on the **B** mechanical sensitivity, **C** open field test, **D** Y-maze test, and **E** novel object preference test. *N* = 5 for the control, PBS, and unsus group, *n* = 10 for the sus group. Tukey’s post hoc tests; *p < 0.05, **p < 0.01. **F** Chaos index, *α* diversity. **G** Principal coordinates analysis (PCoA), *β* diversity. **H** The proportion of dominant species at the genus level (Circos). **I** Common species in the control, PBS-treat, susceptible donors-transferred, and susceptible donor–transferred samples at the genus level. **J** Species with different abundance at the phylum level

Since the CPSP rats with cognitive impairment had an abnormal gut microbiota composition, the fecal microbiota of unsusceptible rats (CPSP rats without cognitive impairment) or susceptible rats (CPSP rats with cognitive impairment) was transferred to germ-free rats (Fig. 3A). After FMT, recipients of susceptible microbiota demonstrated cognitive deficit behavior in the novel object preference test (Fig. 3E), but not in mechanical sensitivity (Fig. 3B), motor ability (Fig. 3C), and Y-maze test (Fig. 3D). These findings show that the fecal microbiota transplantation from cognitively compromised pain rats can induce the hallmarks of cognitive deficit behavior to germ-free recipient rats.

As demonstrated in Fig. 3F, the α-diversity of chaos index decreased significantly in PBS-treat pseudo-germ-free rats because of microbiota depletion. While bacterial α-diversity did not differ between unsusceptible donor–transferred and susceptible donors-transferred pseudo-germ-free rats, the β-diversity (PCoA, *R*^2^ = 0.5417, *p* = 0.001; Fig. 3G) showed notable differences between these groups, which revealed that the transplantation of gut microbiota from cognitive impairment rats induced by pain altered the microbiota composition. The structure of the microbiome was significantly altered after fecal transplantation in rats (Fig. 3H), and SCFA-producing bacteria [37, 38], including *Bacteroidaceae*, *Coprobacillus*, and *Fecalitalea*, were prevalent in control, PBS-treat, and the unsusceptible donor–transferred samples, but absent in the majority of susceptible donor–transferred samples (Fig. 3I and J). These findings show a link between gut microbiome disruptions and cognitive abnormalities in chronic postoperative pain rats.

### SCFA Intake Ameliorated Chronic Postsurgical Pain-Related Cognitive Deficits

**Fig. 4:**
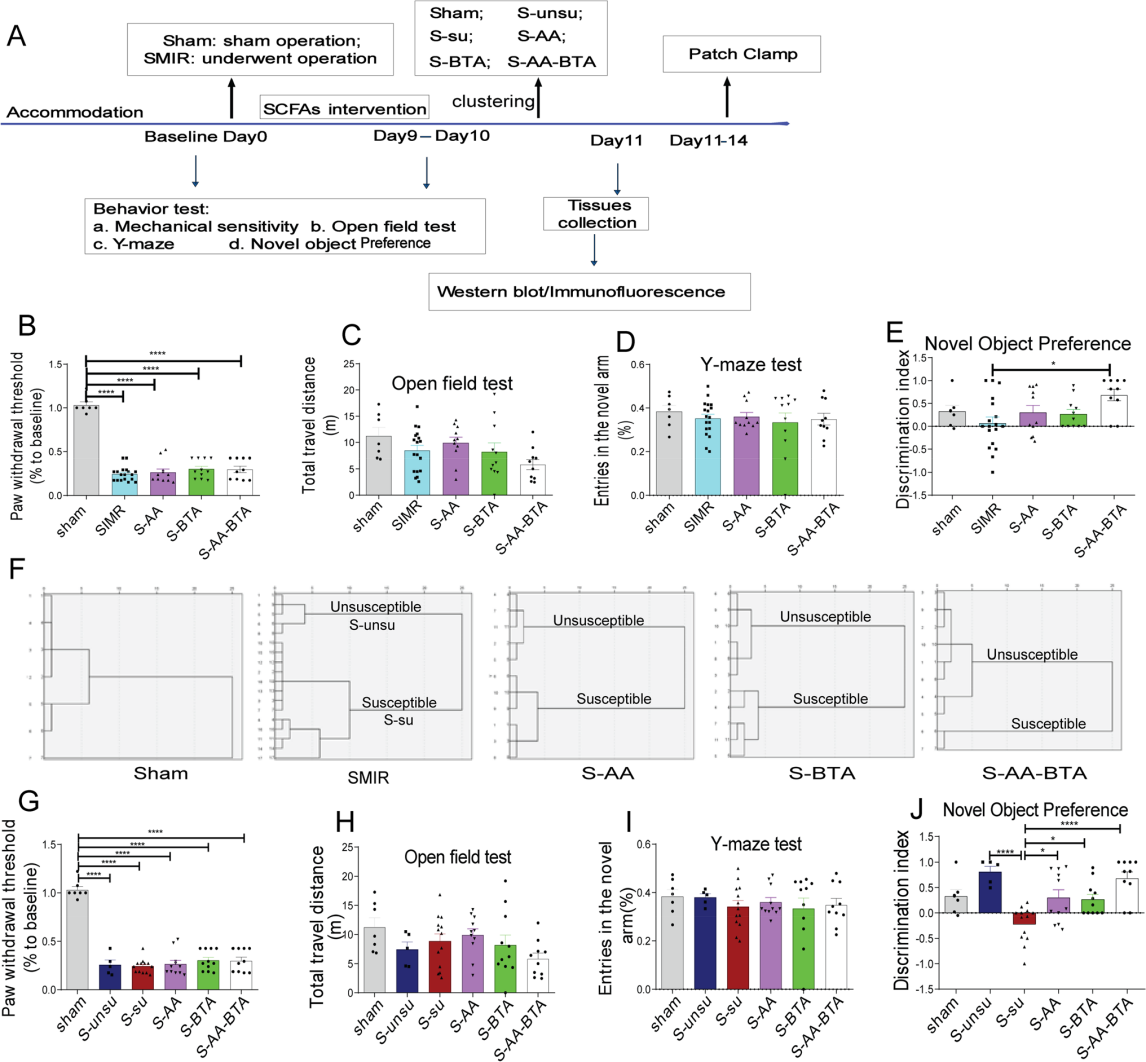
SCFA treatment alleviated pain-induced cognitive deficits in rats. **A** Schematic of the SCFA intervention on the pain-induced cognitive deficits. Behavioral phenotypes after SCFA supplementation (acetate and butyrate), including **B** mechanical sensitivity, **C** the open field test, **D** Y-maze test, and **E** the novel object preference test in the sham group, SMIR group, S-AA group (SMIR rats with acetate), S-BTA group (SMIR rats with butyrate), S-AA-BTA group (SMIR rats with acetate and butyrate). **F** Dendrogram of hierarchical clustering analysis on the Y-maze test and the novel object preference test. sham group, *n* = 7; SMIR group, *n* = 18, rats in SMIR group were divided into S-su group (*n* = 13, SMIR rats with cognitive dysfunction) and S-unsugroup (*n* = 5, SMIR rats without cognitive dysfunction) by hierarchical clustering analysis; S-AA group, *n* = 11; S-BTA group, *n* = 11; S-AA-BTA group, *n* = 10. **G**–**J** Behavioral phenotypes in CPSP rats after hierarchical clustering analysis, including **G** mechanical sensitivity, **H** the open field test, **I** Y-maze, and **J** the novel object preference test. Results are expressed as mean ± SEM; Tukey’s post hoc tests; *p < 0.05, ***p < 0.001

Previous research has revealed that the gut microbiome mediates pain-related cognitive deficits and that the genera that generate SCFAs (acetate and butyrate) were dramatically reduced not only in the susceptible group (Fig. 2) but also in the susceptible-transferred group (Fig. 3). As a result, we postulated that the drop in acetate and butyrate levels in susceptible rats was connected to their cognitive deficiencies. To put this theory to the test, CPSP rats were given acetate and butyrate supplements (Fig. 4A). SCFA supplementation did not influence mechanical sensitivity (Fig. 4B), motor ability (Fig. 4C), or spatial orientation learning memory capacity (Fig. 4D). As can be shown, only acetate and butyrate therapy corrected cognitive deficit behavior in a novel object preference test (Fig. 4E). Surprisingly, the hierarchical clustering analysis (Fig. 4F) revealed that SCFA administration considerably decreased the occurrence of cognitive impairment in the CPSP rats (SMIR group: 72.22%, S-AA group: 54.55%, S-BTA group: 45.46%, S-AA-BTA group: 20%).

Then, by hierarchical clustering, the rats that underwent SMIR surgery were divided into the cognitively normal pain rats (s-unsu group) and cognitively compromised pain rats (s-su group). We next evaluated the difference among these groups. As a result, SCFA therapy considerably enhanced the discrimination index compared to the s-su group (Fig. 4J). In comparison, there was no difference in mechanical sensitivity (Fig. 4G), motor ability (Fig. 4H), or spatial orientation learning memory capacity (Fig. 4I). These findings show that SCFA supplementation can reverse CPSP-induced cognitive impairment.

### SCFA Treatment Regulated Histone Acetylation Through the ACCS2-HDAC2 Axis

**Fig. 5:**
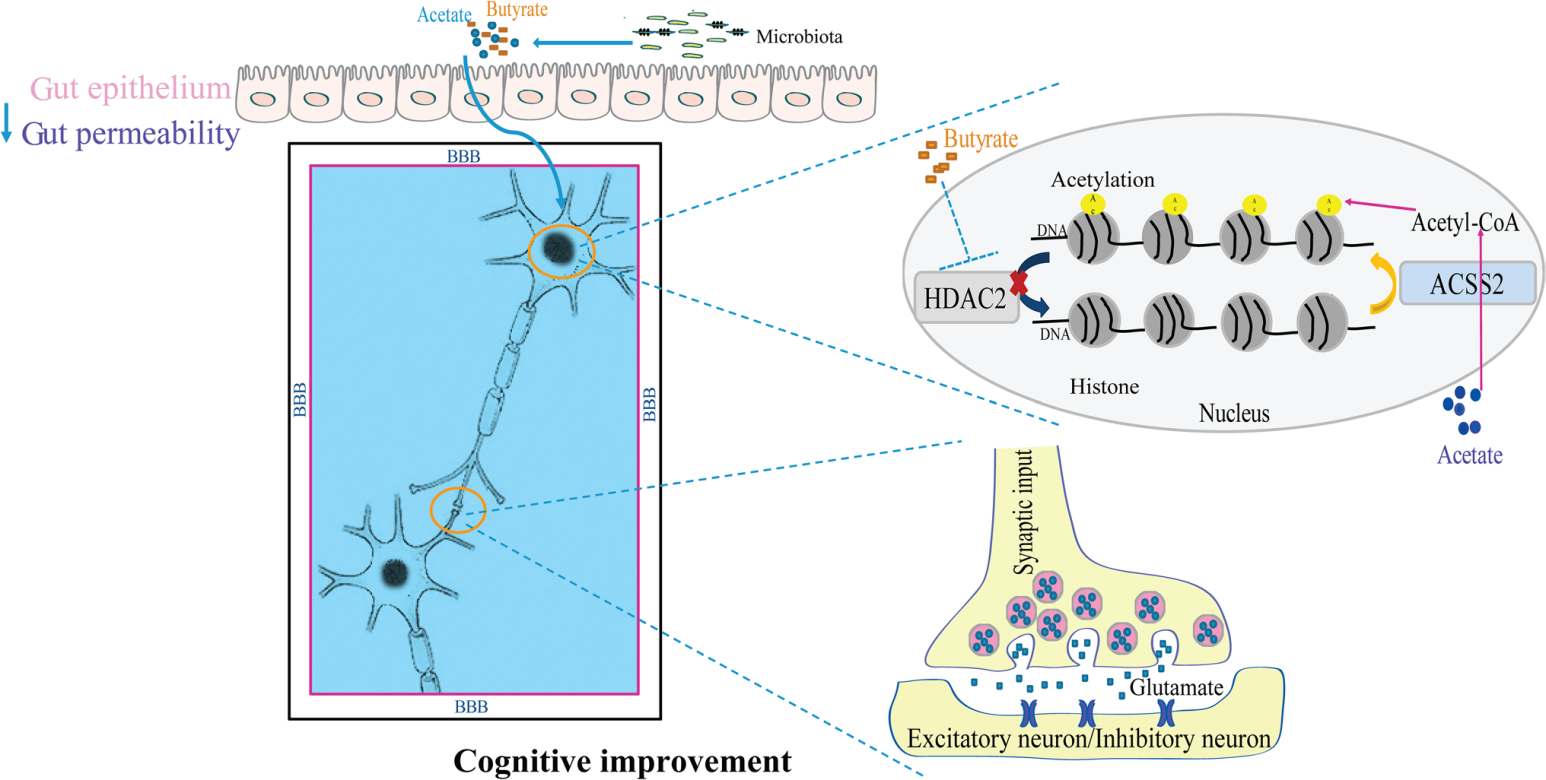
A hypothetical mechanism for SCFAs alleviates pain-related cognitive deficits by improving histone acetylation and synaptic transmission. The gut microbiota metabolites (acetate and butyrate) approach brain tissue by blood flow and reduce permeability to the gut and BBB (blood–brain barrier). In the nucleus, this acetate was converted into acetyl-CoA by ACSS2 (acetyl-CoA synthetase2) to supply carbon for histone acetylation. Butyrate, an HDAC inhibitor, inhibits histone deacetylase activity to increase histone acetylation. Improved histone acetylation restores the excitatory synaptic input to the excitatory and inhibitory neurons in the mPFC, CeA, and hippocampal CA1 areas in the synapses. Then, improved histone acetylation and synaptic transmission alleviate pain-related cognitive deficits

**Fig. 6:**
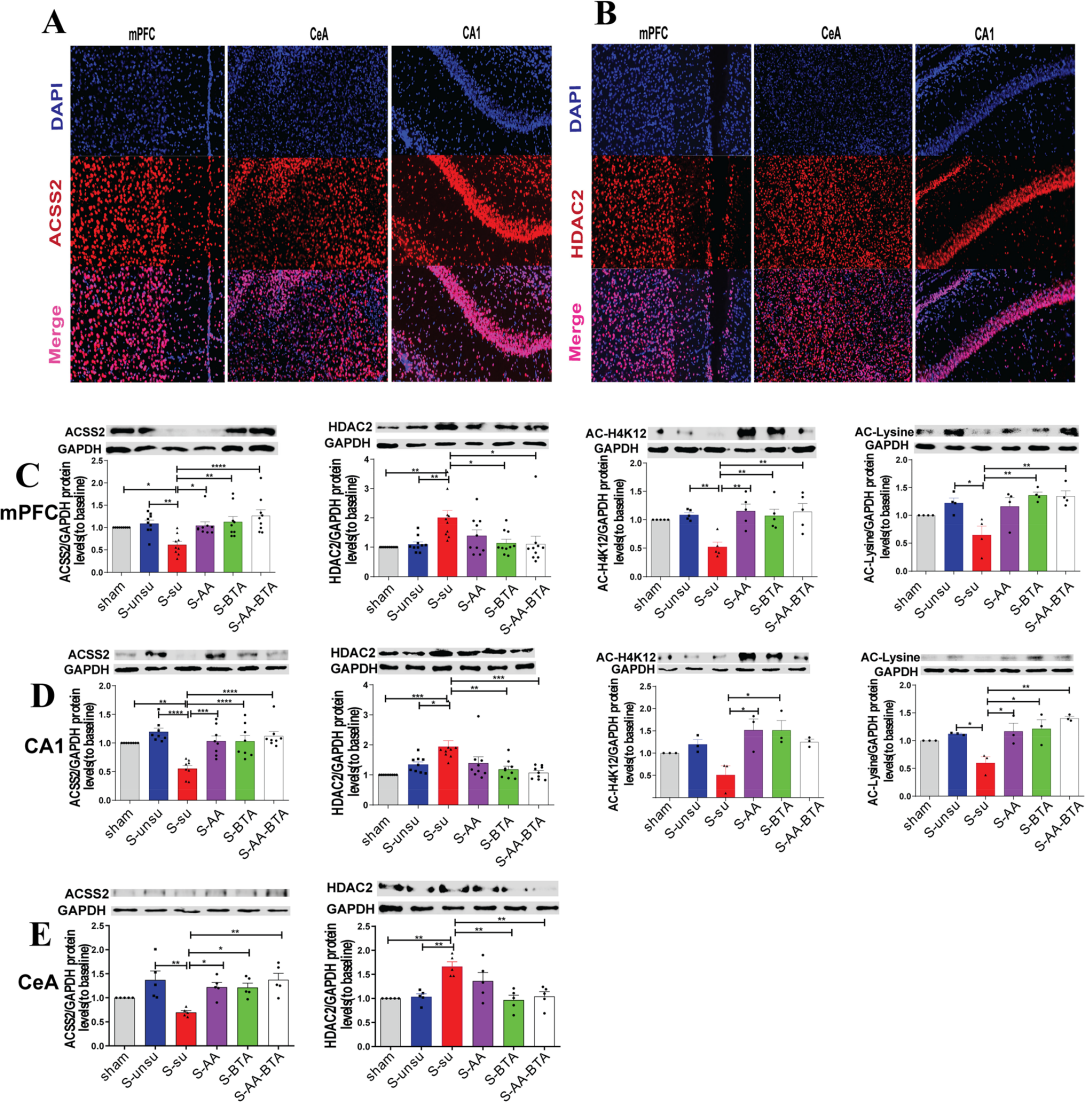
SCFA treatment regulated histone acetylation through the ACCS2-HDAC2 axis. **A**–**B** Representative immunofluorescent images of ACSS2 (red) and HDAC2 (red) in mPFC, CeA, and hippocampal CA1. Nuclei are stained blue. **C** Western blot analysis of ACSS2, HDAC2, ace-H4K12, and ace-lysine in mPFC in rats after SCFA treatment. **D** The expression level of ACSS2, HDAC2, ace-H4K12, and ace-lysine in hippocampal CA1 area after SCFAs treatment. **E** Representative western blots showing ACSS2 and HDAC2 expression in CeA area after SCFA treatment. Results are expressed as mean ± SEM; Tukey’s post hoc tests; *p < 0.05, **p < 0.01, ***p < 0.001, ****p < 0.0001

Sodium butyrate, an HDAC inhibitor, has improved memory via increasing histone acetylation [19]. Acetyl-CoA synthetase 2 (ACSS2) is a direct controller of histone acetylation and spatial memory that employs acetate as a source for nuclear acetyl-CoA synthesis [21]. Anatomically, the hippocampus, central amygdala, and medial prefrontal cortex—all involved in cognitive modulation—are also involved in pain control [8, 39, 40]. Given this, we investigated whether applying direct SCFAs to CPSP rats may ameliorate pain-induced cognitive impairments by increasing histone acetylation via the ACCS2-HDAC2 axis in these regions (Fig. 5). ACSS2 and HDAC2 immunostaining demonstrated that they were located in the nucleus (Fig. 6 A and B), consistent with prior results indicating ACSS2 and HDAC2 are abundantly localized to the nucleus of neurons [18, 22]. In the western blot test, we discovered that HDAC2 expression was dramatically raised, while ACSS2 expression was reduced in the mPFC (Fig. 6C), hippocampal CA1 (Fig. 6D), and CeA areas (Fig. 6E) of the susceptible group, but SCFA therapy restored them to normal levels. In keeping with these findings, we noticed reduced acetylation of H4K12 and lysine in the susceptible group’s mPFC and hippocampal CA1 region. Similarly, supplementing with SCFAs restored H4K12 and lysine acetylation. These data suggest that SCFA administration to CPSP rats alleviates pain-induced cognitive impairments by increasing histone acetylation via the ACCS2-HDAC2 axis.

### SCFA Treatment Restored Synaptic Deficits in Cognitively Compromised Pain Rats

**Fig. 7:**
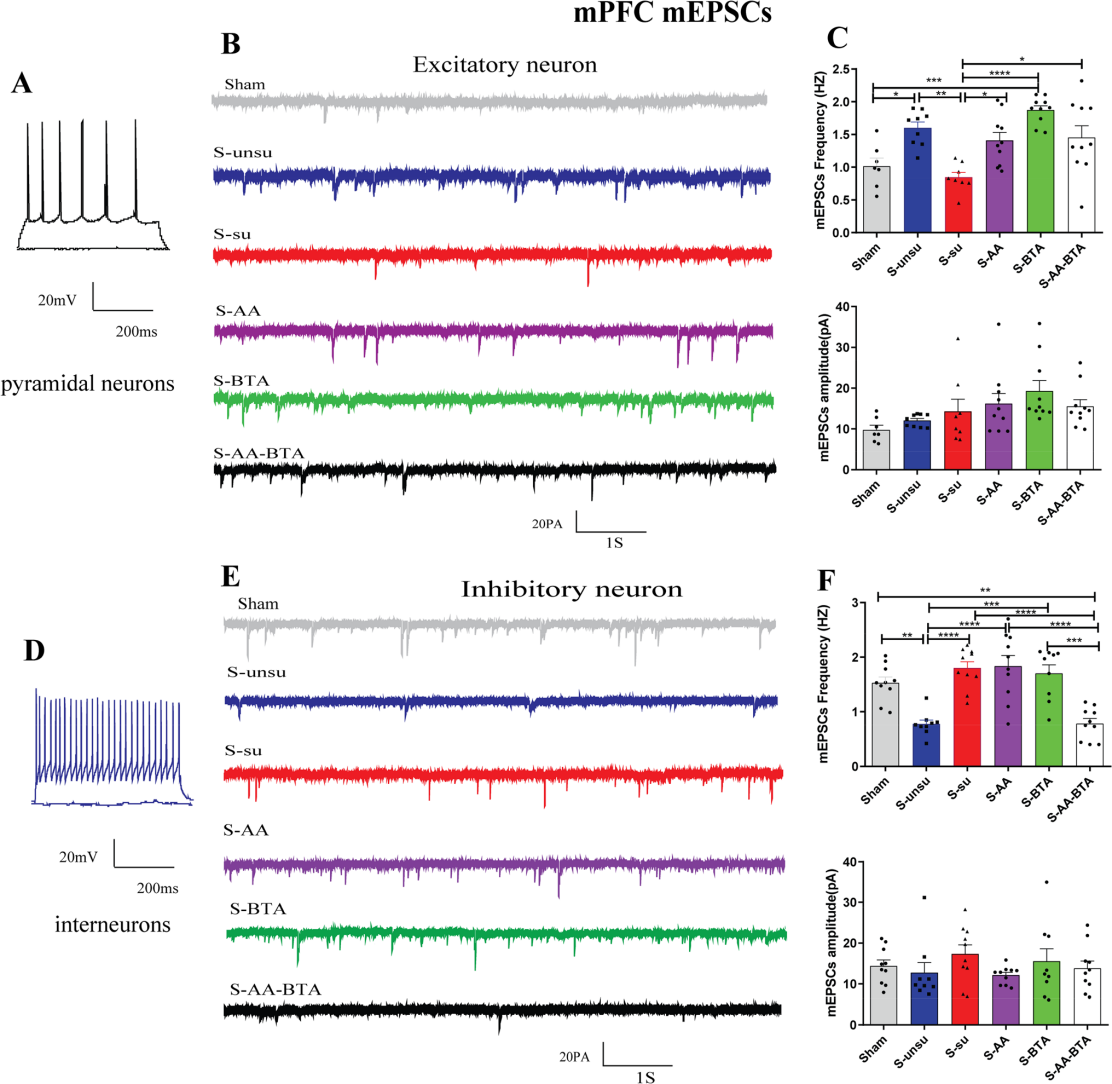
SCFA treatment rescued synaptic deficits in layer II/III of the mPFC neurons in cognitively compromised pain rats. **A**–**C** Effect of the SCFAs intervention on the spontaneous synaptic transmission of excitatory neurons in pain-induced cognitive deficits rats. **A** Current-clamp recordings to identify excitatory neurons in the mPFC slices. **B** Original traces of mEPSCs of excitatory neurons recorded from the six groups. **C** Bar graphs showing the frequency and the amplitude of mEPSCs in mPFC excitatory neurons. *N* = 5 rats for each group. **D**–**F** Spontaneous synaptic transmission of the mPFC inhibitory neurons in slices. **D** Current-clamp recordings to identify inhibitory neurons in the mPFC slices. **E** Original traces of mEPSCs in slices from the six groups. **F** Bar graphs showing the frequency and the amplitude of mEPSCs in mPFC inhibitory neurons. Results are expressed as mean ± SEM; *n* = 5 rats for each group. Tukey’s post hoc tests; *p < 0.05, **p < 0.01, ***p < 0.001, ****p < 0.0001

**Fig. 8:**
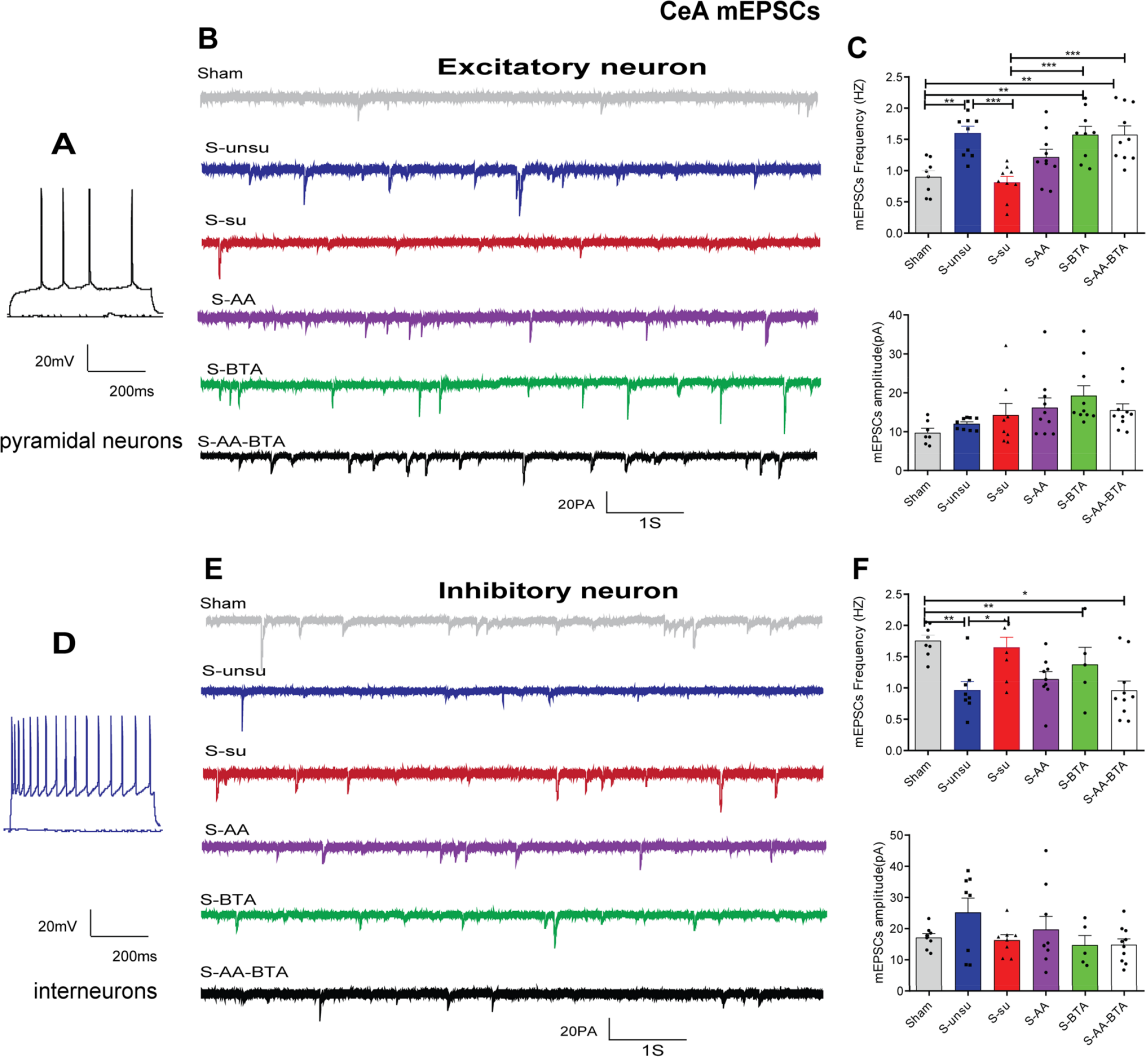
SCFA treatment improved synaptic deficits in CeA neurons in cognitively compromised pain rats. **A**–**C** Effect of the SCFAs intervention on the spontaneous synaptic transmission of excitatory neurons in pain-induced cognitive deficits rats. **A** Current-clamp recordings to identify excitatory neurons in the CeA. **B** Examples of mEPSCs of excitatory neurons recorded from the six groups. **C** Bar graphs showing the frequency and the amplitude of mEPSCs in CeA excitatory neurons. *N* = 5 rats for each group. **D**–**F** Effect of the SCFA intervention on the spontaneous synaptic transmission of CeA inhibitory neurons in slices. **D** Current-clamp recordings to identify inhibitory neurons in the CeA. **E** Examples of mEPSCs of inhibitory neurons recorded from the six groups. **F** Bar graphs showing the frequency and the amplitude of mEPSCs in the inhibitory neurons. Results are expressed as mean ± SEM; *n* = 5 rats for each group. Tukey’s post hoc tests; *p < 0.05, **p < 0.01, ***p < 0.001, ****p < 0.0001

**Fig. 9:**
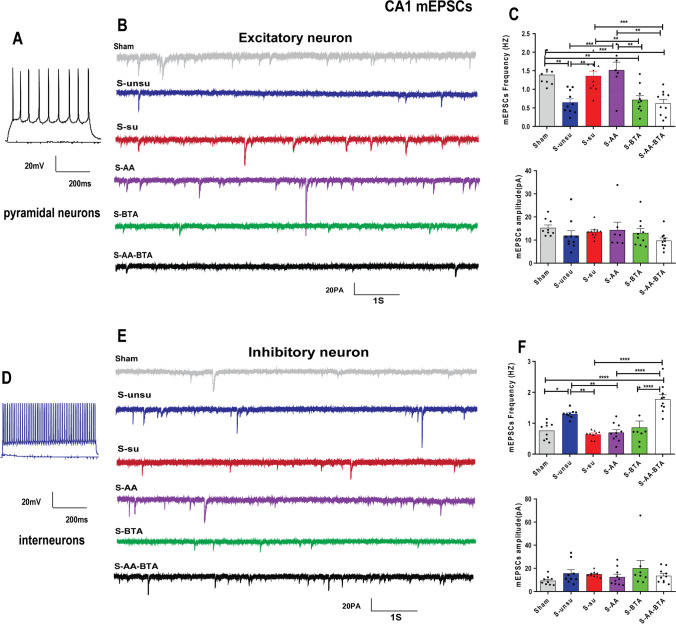
SCFA treatment restored synaptic deficits in hippocampal CA1 neurons in cognitively compromised pain rats. **A**–**C** Effect of the SCFA intervention on the spontaneous synaptic transmission of excitatory neurons in hippocampal CA1 slices neurons. **A** Current-clamp recordings to identify excitatory neurons in the hippocampal CA1. **B** Representative mEPSC recording traces from the six groups. **C** Bar graphs showing the frequency and the amplitude of mEPSCs in hippocampal CA1 excitatory neurons. *N* = 5 rats for each group. **D**–**F** Effect of the SCFA intervention on the spontaneous synaptic transmission of hippocampal CA1 inhibitory neurons. **D** Current-clamp recordings to identify inhibitory neurons in the hippocampal CA1. **E** Representative mEPSCs of inhibitory neurons recording traces from the six groups. **F** Bar graphs showing the frequency and the amplitude of mEPSCs in hippocampal CA1 inhibitory neurons. Results are expressed as mean ± SEM; *n* = 5 rats for each group. Tukey’s post hoc tests; *p < 0.05, **p < 0.01, ***p < 0.001, ****p < 0.0001

Histone acetylation epigenetic alterations have been connected to dendritic spine density and synapse number [17, 18]. SCFA therapy has been shown to improve synaptic function in the hippocampus and PFC, hence restoring maternal obesity-induced cognitive-behavioral impairments in children [9]. The next step was to see if SCFA therapy might reverse synaptic impairments in pain rats with cognitive impairment. Excitatory pyramidal neurons and inhibitory interneurons in mPFC (Fig. 7A and D), CeA (Fig. 8A and D), and hippocampal CA1 (Fig. 9A and D) were identified by accommodating responses to a sustained depolarizing intracellular current stimulation. An examination of miniature excitatory postsynaptic currents (mEPSCs) on mPFC excitatory neurons indicated that the frequency of mEPSCs in the susceptible group was considerably lower than in the unsusceptible group, despite no discernible change in amplitude (Fig. 7B and C). Significantly, acetate and butyrate supplementation increased the frequency of mEPSCs from the mPFC excitatory neurons in the S-AA, S-BTA, and S-AA-BTA groups (Fig. 7E and F).

Given that GABAergic inhibition is essential in controlling pain and cognitive functions [41, 42], we investigated the effect of SCFAs on inhibitory neurons. Consistently, acetate and butyrate supplementation substantially reduced the mEPSC frequency of the mPFC inhibitory neurons in the S-AA-BTA group (Fig. 7E and F). In the susceptible group, however, the frequency of mEPSCs in the mPFC inhibitory neurons was considerably higher than in the unsusceptible group.

We examined at the same time and looked at changes in synaptic transmission in CeA and hippocampal CA1 excitatory and inhibitory neurons. As indicated in Fig. 8B and C, acetate and butyrate supplementation increased the frequency of mEPSCs in CeA excitatory neurons in the S-AA, S-BTA, and S-AA-BTA groups. Surprisingly, SCFA administration did not affect the frequency of mEPSCs in CeA inhibitory neurons (Fig. 8E and F). Furthermore, compared to the unsusceptible group, the susceptible group’s mEPSC frequency of hippocampal CA1 excitatory neurons was considerably higher (Fig. 9B and C). Interestingly, butyrate, but not acetate, reversed synaptic impairments in hippocampal CA1 excitatory neurons in rats with pain-induced cognitive abnormalities. Meanwhile, acetate and butyrate supplementation improved synaptic impairments in hippocampal CA1 inhibitory neurons (Fig. 9E and F). These findings underscore the importance of SCFAs in making chronic postoperative rats resistant to cognitive deficits.

## Discussion

Cognitive impairment is one of the most prevalent postoperative consequences of chronic pain [1–3], but the underlying processes and effective therapies are still unknown. This investigation discovered that about 60% of CPSP rats had cognitive impairment, which was consistent with earlier clinical studies [43, 44]. Furthermore, we discovered that CPSP rats with cognitive impairment had an aberrant composition of gut microbiota and a lower number of SCFA-producing bacteria using 16S rRNA sequencing of microbiota. Then, gut microbiota transplantation from cognitively impaired CPSP rats was demonstrated to transmit cognitive impairment behavior. These findings suggested a link between gut microbiota composition and cognitive-behavioral alterations.

Due to the lower number of SCFA-producing bacteria in susceptible donors and susceptible donor-transferred recipient rats, SCFAs, primarily acetate and butyrate, were supplied further. Then, it comes as little surprise that SCFA therapy reduced pain-induced cognitive impairments in rats. In search of molecular reasons for the beneficial intervention, we discovered that SCFA administration reversed histone acetylation and synaptic impairments in mPFC, CeA, and hippocampal CA1 neurons in rats with pain-induced cognitive deficiencies via the ACCS2-HDAC2 axis. Short-chain fatty acids (SCFAs), which are major metabolites of the microbiota during anaerobic fermentation, may help to alleviate persistent postsurgical pain-induced cognitive impairments by increasing histone acetylation and synaptic transmission. It is worth noting that we found no difference in pain threshold following gut microbiota colonization and SCFA therapy. However, experimental investigations have shown that a histone deacetylase inhibitor reduced neuropathic pain by increasing histone acetylation. This disparity might be attributed to the study’s use of the non-neuropathic pain model [45].

The microbiota has previously been demonstrated to regulate cognitive function [9, 10], and mother-to-offspring transmission of the gut microbiome has been shown to mediate maternal high-fat diet-induced learning and memory problems in offspring [9]. Furthermore, fecal microbiota transplantation has been shown to enhance cognitive performance and lower liver function indices in rats with hepatic encephalopathy [46]. In line with this, we demonstrated in the current work that transplanting the gut microbiota from CPSP rats with cognitive impairment transmitted cognitive impairment behavior. We also discovered a reduction in the number of SCFA-producing bacteria in susceptible donors and susceptible donor-transferred recipient rats. These findings were in harmony with studies by Liu and Ragusa [9, 47].

Interestingly, the butyrate concentration was greater in the CPSP rats with cognitive impairment, despite no significant difference in other SCFAs across groups. Notably, any gut microbe-brain interaction must pass through two barriers (the gut epithelial barrier and the blood–brain barrier), and permeability through these barriers has been demonstrated to be affected by the microbiome [11, 14]. SCFAs, main butyrate, have been shown to strengthen the integrity of the epithelial barrier and the blood–brain barrier [14]. As a result, it is reasonable to speculate that the rise in butyrate in the serum was caused by increased intestinal permeability rather than the excessive fermentation reported in CPSP rats with cognitive impairment [48]. Of course, whether the increase in butyrate in the serum was caused by increased intestinal permeability and excessive fermentation has to be investigated further.

Histone acetylation epigenetic changes have been shown to have a crucial function in numerous cognitive processes due to their pivotal involvement in neural development [49]. Acetylation and deacetylation are required for proper acetylation. HDAC inhibitors or HDAC2 knock-down have been proven to ameliorate memory dysfunction in mice with HDAC2 overexpression [50]. It was shown that lipid-derived acetyl-CoA, which may be formed from SCFAs catalyzed by acetyl-CoA synthetase 2 (ACSS2), is a genuine source (up to 90%) of carbon for histone acetylation, even in the presence of abundant glucose, using ^13^C-carbon tracing and acetyl-proteomics histone acetylation [20, 21]. Given that ACSS2 requires acetate for nuclear Acetyl-CoA production and sodium butyrate is an HDAC inhibitor, it is highly plausible that acetate and butyrate supplementation may improve cognitive impairments via increasing histone acetylation. In keeping with this idea, we discovered that SCFA therapy restored HDAC2 and ACSS2 expression to normal levels.

Similarly, SCFA supplementation improved H4K12 and lysine acetylation, both related to learning and memory [18, 51]. Notably, the supplement of butyrate, an HDAC inhibitor, restored not only HDAC2 but also ACSS2 expression in this study. These findings might be explained in part by the fact that butyrate can be catalyzed by ACSS2 into butyryl-CoA, which plays a critical role in immunological homeostasis maintenance [52].

Acetylation of lysine is critical for synaptic plasticity, and histone acetylation level has been shown to calibrate the correct number of released vesicles and synaptic transmission [53]. Upregulation of histone deacetylase 2 (HDAC2) activity has been associated with dendritic spine density, synapse number, and expression of various cognitive-related genes [17, 18]. Our electrophysiological recordings in brain slices revealed variations in the frequency of mEPSCs in excitatory and inhibitory neurons from the rats with cognitive dysfunction, indicating changes in the number of vesicles released. Meanwhile, SCFA supplementation restored these alterations. These results are consistent with previous studies suggesting HDAC2’s epigenetic regulation of excitatory and inhibitory synaptic transmission played a vital role in modulating cognitive impairment [54]. Thus, the influence of SCFA supplementation on synaptic transmission may be due to the correct number of released vesicles mediated by appropriate acetylation. It is worth noting that the bulk of acetate metabolism in the brain happens in astrocytes, with neurons accounting for just around 30% of the total [21, 55]. As a result, additional research is needed to investigate the role of astrocytes in the development of pain-induced cognitive impairment. Another primary question was that we found synaptic transmission differences between pain animals with cognitive impairments and those without; however, no differences were detected between cognitively affected pain animals and sham controls. This surprising fact might be attributed to the fact that chronic pain and cognitive dysfunction may have opposite relations with the synaptic transmission in the central nervous system. The literature consistently showed a positive correlation between synaptic transmission and pain [56–59], while a negative correlation between synaptic transmission and cognitive impairment in variable models [60–63].

## Conclusions

To summarize, our findings show that pain-induced cognitive impairments are caused by changes in the makeup of the gut microbiota, and SCFA therapy alleviates pain-induced cognitive deficits by improving histone acetylation and synaptic transmission in brain. It may provide a more detailed knowledge of the underlying epigenetic mechanisms that link gut microbiota, histone acetylation, and pain-related cognitive impairments and suggest that SCFAs might be a viable non-invasive and tractable therapy for cognitive deficiencies. Overall, this finding opens up a new research avenue into preemptive therapies for pain-related cognitive impairments and supports the use of SCFA-dependent perioperative interventions for postoperative rehabilitation in the clinic.

## Data Availability

All relevant data supporting the findings of this study are available from the corresponding author on reasonable request.

## Abbreviations

ACSS2: Acetyl-CoA synthetase2
CeA: Central amygdala
CPSP: Chronic postsurgical pain
FMT: Faecal microbial transplantation
HDAC2: Histone deacetylase 2
mEPSCs: Miniature excitatory postsynaptic currents
mPFC: Medial prefrontal cortex
PCoA: Principal co-ordinate analysis
PWL: Paw withdrawal threshold
SCFAs: Short-chain fatty acids
SMIR: Skin/muscle incision and retraction
